# Loss of *Msh2* and a single-radiation hit induce common, genome-wide, and persistent epigenetic changes in the intestine

**DOI:** 10.1186/s13148-019-0639-8

**Published:** 2019-04-27

**Authors:** Maria Herberg, Susann Siebert, Marianne Quaas, Torsten Thalheim, Karen Rother, Michelle Hussong, Janine Altmüller, Christiane Kerner, Joerg Galle, Michal R. Schweiger, Gabriela Aust

**Affiliations:** 1 Interdisciplinary Center for Bioinformatics (IZBI), Leipzig University, Leipzig, Germany; 2 Laboratory for Translational Epigenetics and Tumor Genetics, University Hospital Cologne, Cologne, Germany; 3 Graduate School for Biological Sciences (GSfBS), University of Cologne, Cologne, Germany; 4 Department of Surgery, Research Laboratories, Leipzig University, Liebigstr. 19, D-04103 Leipzig, Germany; 5 Laboratory for Clinical and Experimental Hepatology (LCEHep) Section of Hepatology, Clinic for Gastroenterology and Rheumatology, University Hospital Leipzig, Leipzig, Germany; 6 Center for Molecular Medicine Cologne, University of Cologne, Cologne, Germany; 7 Cologne Center for Genomics, University of Cologne, Cologne, Germany

**Keywords:** Intestine, Mismatch repair deficiency, Radiation, Msh2, Histone H3 methylation

## Abstract

**Background:**

Mismatch repair (MMR)-deficiency increases the risk of colorectal tumorigenesis. To determine whether the tumors develop on a normal or disturbed epigenetic background and how radiation affects this, we quantified genome-wide histone H3 methylation profiles in macroscopic normal intestinal tissue of young radiated and untreated MMR-deficient *VCMsh2*^*LoxP/LoxP*^ (*Msh2*^*−/−*^) mice months before tumor onset.

**Results:**

Histone H3 methylation increases in *Msh2*^*−/−*^ compared to control *Msh2*^*+/+*^ mice. Activating H3K4me3 and H3K36me3 histone marks frequently accumulate at genes that are H3K27me3 or H3K4me3 modified in *Msh2*^*+/+*^ mice, respectively. The genes recruiting H3K36me3 enrich in gene sets associated with DNA repair, RNA processing, and ribosome biogenesis that become transcriptionally upregulated in the developing tumors. A similar epigenetic effect is present in *Msh2*^*+/+*^ mice 4 weeks after a single-radiation hit, whereas radiation of *Msh2*^*−/−*^ mice left their histone methylation profiles almost unchanged.

**Conclusions:**

MMR deficiency results in genome-wide changes in histone H3 methylation profiles preceding tumor development. Similar changes constitute a persistent epigenetic signature of radiation-induced DNA damage.

**Electronic supplementary material:**

The online version of this article (10.1186/s13148-019-0639-8) contains supplementary material, which is available to authorized users.

## Introduction

Mismatch repair (MMR) deficiency originates from loss of function of MMR proteins. Germline alterations of MMR genes have been related to Lynch syndrome [1] which is associated with a predisposition to a variety of malignant tumors including colorectal cancer. About 10% to 15% of all colorectal cancers are MMR-deficient. *MSH2* and *MLH1* are the genes that are most commonly altered [2, 3], and a loss of function results in an immediate increase of mismatch mutations [4] which accumulate particularly in microsatellites giving rise to the phenomenon of microsatellite instability (MSI) [2, 5].

The *VCMsh2*^*LoxP/LoxP*^ (*Msh2*^*−/−*^) mouse is a suitable model to quantify the severity and time course of molecular alterations caused by MMR deficiency [6, 7]. In organoids derived from intestinal stem cells (ISC) of these mice, MSI increases continuously from birth and affects microsatellites in all organoids already at a mouse age of 2 months. However, intestinal carcinomas become detectable only at a mouse age of 12 months and their occurrence is paralleled by tissue-wide changes in gene expression [7]. This obvious time delay between the early MSI in organoids and the late tumor onset as well as tissue-wide transcriptional changes suggest additional, as yet hidden, (epi)genetic changes.

Among the epigenetic changes frequently associated with tumor formation are histone methylations [8]. Depending on the lysine which is methylated, they can induce transcriptional activation or repression [9]. Tri-methylation of histone H3 at lysine 4 (H3K4me3) facilitates initiation of gene transcription, while H3K36me3 stabilizes elongation. H3K27me3 is frequently associated with suppression of gene transcription. To clarify whether histone methylation changes are already present in young *Msh2*^*−/−*^ mice, and whether they are capable of inducing the tissue-wide transcriptional changes present in aged *Msh2*^*−/−*^ mice [7], we performed genome-wide chromatin immunoprecipitation with high-throughput sequencing (ChIP-seq) comparing 4-month-old *Msh2*^*−/−*^ and control *Msh2*^*+/+*^ mice.

Based on our results, the question arose whether the observed changes are associated with loci-specific DNA repair or are part of a response on genomic instability at the cell level. Therefore, we analyzed whether a similar epigenetic response can be induced in the intestine by a single-radiation hit. ChIP-sequencing (ChIP-seq) analysis was performed on purpose 4 weeks after radiation when short term transcriptional and epigenetic responses are abated [10, 11]. At this time point, most intestinal crypts had already undergone monoclonal conversion, that is, they had become populated by the progeny of a single ISC, while all other ISC and their clones had vanished due to competition for niche space [12]. Potential selection of stress-tolerant ISC is mostly completed by clonal competition [13]. Moreover, these measurements enable us to answer the question of whether a single-radiation hit induces additional epigenetic changes in MMR-deficient tissue, which is controversially discussed at the gene level [14].

By comparing the epigenetic changes induced by loss of *Msh2* and a single-radiation hit, we identified a common, genome-wide, and persistent epigenetic response.

## Materials and methods

### Mouse and human tissue

The mouse strains B6.SJL-Tg(Villin-cre)997Gum/J and B6.Cg-*Msh2*^*tm2.1Rak*^/J were obtained from The Jackson Laboratory (Bar Harbor, USA). By crossing both, the conditional Msh2 allele has been placed under the control of the Villin-Cre transgene. Mice were genotyped as described [6]. *VC*^*+/?*^*Msh2*^*LoxP/LoxP*^ (*Msh2*^*−/−*^) and *VC*^*−/−*^*Msh2*^*LoxP/LoxP*^ (*Msh2*^*+/+*^) mice were bred under specific pathogen-free conditions. A mouse doublet consisted of one *Msh2*^*−/−*^ and one *Msh2*^*+/+*^ mouse from one litter. In addition, we analyzed normal human colon samples of patient 7 (74 years old, female, *MSH6* mutation carrier) of the cohort described in [3].

### Radiation of mice and collection of tissue samples

Three-month-old mice were placed in the radiation unit (X-ray generator, Gulmay D3225, round tube 170 mm, radiation level at 2.5 cm: 1.068 Gy/min). One pair of mice of each genotype was irradiated with a total dose of 0.5 Gy, while the other pair was left non-radiated. All mice were killed after 28 days. Samples of the proximal jejunum were dissected, shock-frozen without delay, and stored in liquid nitrogen until further use.

### ChIP

Chromatin was prepared from up to 50 mg jejunum using SDS Shearing Buffer and the truChlP Tissue Chromatin Shearing Kit (Covaris, Brighton, UK). Chromatin immunoprecipitations (ChIPs) were run on the IP-Star compact system using the Auto iDeal ChIP-seq kit for histones (Diagenode, Seraing, Belgium). For ChIP-seq validation, shredded intestinal tissue was cross-linked in 1% formaldehyde for 8 min at room temperature and homogenized in ZR 2.0 mm BashingBead™ Lysis Tubes (Zymo Research Corp., Tustin, USA) with a Precellys24 homogenization device (VWR International GmbH, Darmstadt, Germany). ChIP assays were performed with minor modifications as described [15]. DNA from 15 mg tissue was used in each IP with H3K4me3, H3K27me3, H3K36me3, and H3pan premium rabbit polyclonal antibodies (Diagenode). A non-targeting IgG rabbit antibody (Cell Signaling, Leiden, Netherlands) was used to control non-specific signals. One microliter of purified chromatin was applied for semi-quantitative real-time PCR using the absolute qPCR SYBR Green Mix (Thermo) performed with the ABI 7500 Real-Time PCR System (Applied Biosystems, Forster City, USA). Primers are given in the Additional file 1. All PCR results were normalized to input controls and to an unmodified region (background).

### Quantitative PCR and Illumina sequencing

As quality control for ChIP precipitates, a quantitative real-time PCR analysis was performed in a 384-well format in 10 μl volume using the GoTaq qPCR Master Mix (Promega, Mannheim, Germany) and run on a LightCycler 480 (Roche Diagnostics, Mannheim, Germany). Primers are given in the Additional file 1. The relative enrichment was calculated using the % input method. Library preparations were performed according to the TrueSeq LT PCR free or TruSeq Nano DNA Kit instructions (Illumina, San Diego, USA). Sequencing was performed on the Illumina HiSeq 4000 with 76 bp paired-end sequencings.

### Data pre-processing and peak calling

The sequenced reads underwent a quality trimming using Cutadapt [16] to ensure a minimum read length of 40 bases and a Phred quality score above 20. Quality reports were generated and analyzed using Fastqc [17]. The high-quality reads were mapped to the mouse reference genome NCBI37/mm9 (GRCh37/hg19) using the software tool segemehl 0.2.0 [18]. Histone modification peaks, i.e., genomic regions, in which modifications are enriched, were identified by applying MACS 1.4.2 [19, 20] with the H3pan data as controls and the following, modification independent parameter settings: --nomodel -w --space = 30 --bw 300 --pvalue 1e-5. The parameter --shiftsize has been set to 200 for sharp (H3K4me3) and to 600 for broad (H3K27me3, H3K36me3) peaks.

Published H3K4me3, H3K27me3, H3K36me3, and DNA methylation sequencing data have been reanalyzed using read data provided [21] and calling peaks as described or using the peak data provided [22] directly. In the case of RNA-seq data [3, 22], the raw counts provided were normalized to reads per kilobase million (RPKM) values and transformed into log-scale for further analysis [23]. Accession numbers are provided at the end of this manuscript.

### ChIP-seq data analysis

We generated summarized quality peak lists for each genotype and treatment. Each list contains the peaks that are either consistently detected in both replicates or have a high reliability (MACS fold enrichment > 5.0). For peaks detected in both replicates the maximum peak breadth and tag density was taken. Subsequently, we identified peaks within the promoter region of genes (defined as transcriptional start site,  ±  1000 bases) and the gene bodies (defined as the region between the transcriptional start site and the last base of the gene). The RefSeq gene reference lists for mouse and human were taken from UCSC Table Browser. To avoid gender-specific artifacts, we excluded genes and peaks of the X- and Y-chromosome from the analysis. If a peak had a minimum overlap of 5% with a promoter region and/or a gene body, the respective gene-associated histones were considered to carry the modification in a binary present or non-present manner. Mouse and human data were compared after filtering for conserved genes using BioMarts [Ensembl release 95] [24]. Gene set enrichment analysis was performed using the gene ontology enrichment analysis tool PANTHER Version 12.0 [25, 26]. Data analysis and images were conducted using the statistic software R [3.4.4] (https://www.r-project.org/) [27]. In addition, the data were analyzed by the self-organizing map (SOM)-technique using the R package oposSOM [2.0.0] [28].

### Microarray analysis

mRNA from intestine samples of a 4-month-old radiated and untreated *Msh2*^*+/+*^ and *Msh2*^*−/−*^ mice was analyzed with the Bead Chip Array MouseRef-8 v2 (Illumina, San Diego, USA) [7]. Data were selected that had a *p* value < 5% from the background corrected raw data and subject to quantile normalization. Further analysis was performed together with similar data on 12-month-old untreated *Msh2*^*+/+*^ and *Msh2*^*−/−*^ mice and tumors harvested from the *Msh2*^*−/−*^ mice [7] using the SOM-technique (see above).

## Results

**Fig. 1:**
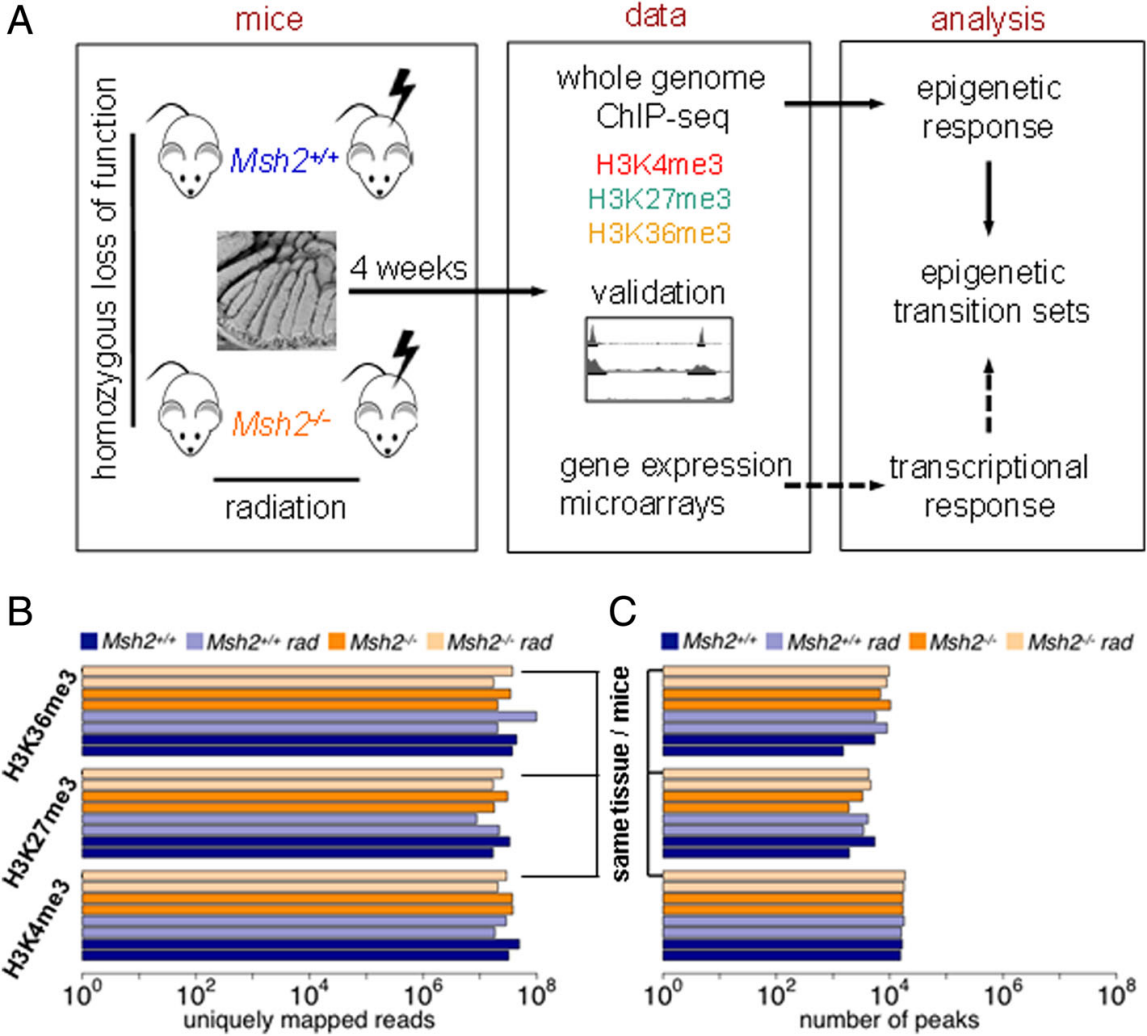
Study design and basic data. **a** Two pairs of non-radiated and 0.5 Gy radiated *Msh2*^*+/+*^ and *Msh2*^*−/−*^ mice were used to study epigenetic changes following *Msh2* loss and radiation. Duplicates of jejunal samples were subjected to whole-genome ChIP-seq analysis of H3K4me3, H3K27me3, and H3K36me3. In parallel, gene expression of these mice was analyzed to characterize the expression of gene sets that show genotype-specific histone methylation states. **b** Number of quality reads uniquely mapped to the mouse genome for the individual samples. **c** Number of peaks called for the individual mouse samples

To characterize the histone modification profiles of the intestine of 4-month-old mice, we measured the histone marks H3K4me3, H3K27me3, H3K36me3, and control H3pan [29] of four doublets of mice. The study design is summarized in Fig. 1a. The number of mapped high-quality reads is shown in Fig. 1b. Based on these data, we constructed summary lists of the genomic regions in which modified histones were detected for each modification and mouse pair. The modified regions are hereinafter referred to as peaks. The number of peaks measured in the individual mice is shown in Fig. 1c. For further analysis, quality peaks have been selected (see “Materials and Methods” trump). Details about the peak breadths and read densities are provided in the Additional file 2.

### Histone methylation is enhanced in *Msh2*^*−/−*^ mice

**Fig. 2:**
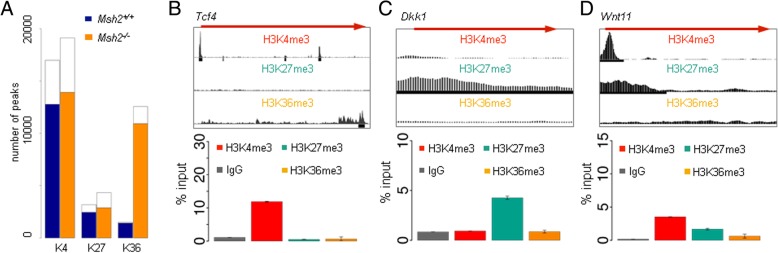
Identification and validation of gene-associated histone modification peaks. **a** Barplots of the total number of quality peaks and the fraction of these peaks associated with genes (colored fraction) for each modification and *Msh2* genotype (K4: H3K4me3, K27: H3K27me3, K36: H3K36me3). **b**–**d** Upper row: screenshots of the H3K4me3, H3K27me3, and H3K36me3 signals from the ChIP-seq analysis displayed in the UCSC Genome Browser (https://genome.ucsc.edu/) at selected genes (position indicated by red arrows) for *Msh2*^*+/+*^ mice. The peak regions are depicted as black bars below the signals. Lower row: ChIP-qPCR for selected genes performed on *Msh2*^*+/+*^ replicates. All PCR results were normalized to input controls and to an unmodified gene region (mean ± SD, *n* = 4)

In total, *Msh2*^*−/−*^ mice show a higher number of peaks, i.e., methylated histones, compared to control mice (peak numbers: 35982 *Msh2*^*−/−*^*,* 21650 *Msh2*^*+/+*^). Independent of the mouse genotype most of the analyzed histone marks are associated with genes (Fig. 2a), i.e., their peaks overlap with promoter and/or gene body regions (91.7% *Msh2*^*−/−*^*,* 72.9% *Msh2*^*+/+*^). The total numbers of H3K4me3 and H3K27me3 gene-associated peaks are similar, while the number of H3K36me3 modified genes is higher in *Msh2*^*−/−*^ mice compared to *Msh2*^*+/+*^ mice.

To validate our results, we examined the histone modification state of selected genes using ChIP-qPCR. Examples are shown in Fig. 2b–d (*Msh2*^*+/+*^ mice) and Additional file 3 (*Msh2*^*−/−*^ mice). We confirmed exclusive enrichment for H3K4me3 in the promoter region of *Tcf4*, a transcriptional effector of Wnt signaling (Fig. 2b), and for H3K27me3 in the promoter region of *Dkk1*, a Wnt antagonist (Fig. 2c). *Wnt11* was found to be bivalent H3K4me3-H3K27me3 modified (Fig. 2d).

### Genes exhibit distinctive histone modification states in *Msh2*^*−/−*^ and *Msh2*^*+/+*^ mice

**Fig. 3:**
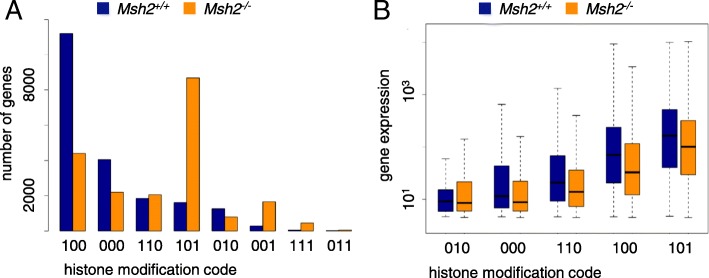
Characterization of gene-associated histone modification states. **a** The numbers of genes associated with a particular histone modification state (0/1-coded, sequence: H3K4me3 H3K27me3 H3K36me3) for both mouse genotypes. The states are ordered decreasingly according to their frequency in *Msh2*^*+/+*^ mice. The most frequent histone modification state of genes in *Msh2*^*+/+*^ and *Msh2*^*−/−*^ mice are the modifications [100] and [101], respectively. The modifications [111] and [011] are rare in both genotypes in agreement with [53]. **b** Boxplots of the expression levels of genes associated with a particular histone modification state for a *Msh2*^*+/+*^ and a *Msh2*^*−/−*^ mouse

Because multiple histone methylations can cause combinatorial effects [30], we determined the histone modification state of all genes. Therefore, each gene obtained a 0/1-triplet code depending on whether the associated histones carry a particular modification (1) or not (0). The code sequence is [H3K4me3 H3K27me3 H3K36me3]. *Msh2*^*−/−*^ clearly differed from *Msh2*^*+/+*^ mice in their histone modification profiles. The numbers of genes associated with a particular histone modification state are shown in Fig. 3a. Genes with a signature of actively and stably transcribed genes, i.e., H3K4me3-H3K36me3 modified genes (signature [101]) are increased in *Msh2*^*−/−*^ compared with *Msh2*^*+/+*^ mice (42.71% vs. 7.95%).

Correlating the histone modification state with the expression of genes, we confirmed well-known relationships (Fig. 3b). The highest mean expression is observed for H3K4me3-H3K36me3 genes [101]. Genes with promoters carrying an H3K4me3 modification [100] show a higher expression than genes with an H3K27me3 modified promoter [010]. Unmodified [000] and bivalent H3K4me3-H3K27me3 genes [110] have an intermediate expression [31].

### Large sets of genes become epigenetically activated in *Msh2*^*−/−*^ mice

**Fig. 4:**
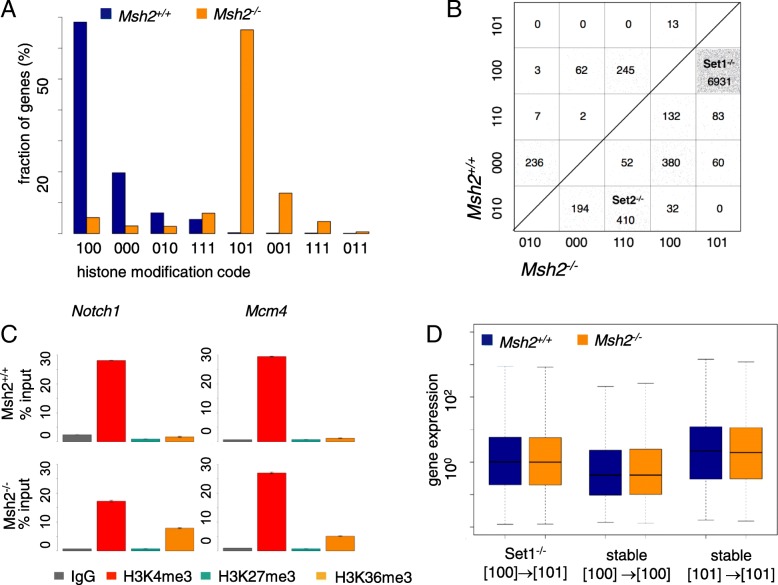
Differential histone methylation in *Msh2*^*−/−*^ compared to *Msh2*^*+/+*^ mice. **a** Comparison of the histone modification state distributions for differentially modified genes (10,767 genes). **b** Transition matrices for the five most common histone modification states. The numbers of genes contributing to the individual transitions are given. The selected state transitions refer to 68% of the differentially modified genes. Notably, each gene below the diagonal becomes epigenetically activated, while genes above the diagonal become epigenetically repressed. **c** ChIP-qPCR for genes of the Set1^−/−^ transition gene sets validating their H3K36me3 recruitment in *Msh2*^*−/−*^ mice. All PCR results were normalized to input controls and to an unmodified gene region (mean ± SD, *n* = 4). **d** Boxplot of the average expression of Set1^−/−^ genes. Set1^−/−^ genes show an intermediate expression between stable [100] and stable [101] modified genes. No expression differences are detected for all sets between *Msh2*^*+/+*^ and *Msh2*^*−/−*^ mice

Among the genes that are differentially modified between both genotypes (10,767 genes), in *Msh2*^*+/+*^ mice genes with no modification [000] or H3K4me3 only [100] are the most frequent, whereas in *Msh2*^*−/−*^ mice H3K4me3-H3K36me3 [101] modified genes are most common (Fig. 4a). To provide information on the number and direction of gene-specific epigenetic changes between *Msh2*^*+/+*^ and *Msh2*^*−/−*^ mice, we calculated a transition matrix (Fig. 4b). We arranged the five most frequent histone modification states in this matrix ascending from epigenetic repression [010] to stable activation [101]. Each square in the matrix comprises genes which show the same histone modification change between *Msh2*^*+/+*^ and *Msh2*^*−/−*^ mice. In the following, we refer to these genes as “transition sets” denoted by XYZ → X’Y’Z’.

We detected two large transition sets by comparing *Msh2*^*+/+*^ and *Msh2*^−/−^ mice. H3K36me3 recruitment is frequently observed in genes that are associated with histones carrying only an H3K4me3 mark in *Msh2*^*+/+*^ mice (100 → 101, 6931 genes; transition gene set 1 for transition *Msh2*^*+/+*^ to *Msh2*^−/−^, in short Set1^−/−^), while recruitment of H3K4me3 is mainly detected for genes associated with histones carrying only a H3K27me3 modification in *Msh2*^*+/+*^ mice (010 → 110, 410 genes; Set2^−/−^).

For selected genes the histone modification differences were exemplarily validated using ChIP-qPCR (Fig. 4c). As in ChIP-seq, *Mcm4* and *Notch1* show an increased level of H3K36me3 in *Msh2*^*−/−*^ compared to *Msh2*^*+/+*^ mice. These genes are essential for the initiation of replication and lineage specification of the progeny of ISC, respectively.

Because recruitment of the H3K36me3 histone methyltransferase Setd2 is RNA polymerase II-dependent [32], we tested whether Set1^−/−^ genes are differentially expressed between *Msh2*^*+/+*^ and *Msh2*^*−/−*^ mice. Surprisingly, the expression of these genes is similar suggesting another way of H3K36me3 enrichment (Fig. 4d). Set1^−/−^ genes are more highly expressed in the *Msh2*^*+/+*^ intestine than those which are [100] modified in both mice. Due to the low expression of H3K27me3 genes, we could not verify Set2^−/−^ genes for their expression.

### A single-radiation hit causes an epigenetic activation in *Msh2*^*+/+*^ mice similar to *Msh2*-loss

**Fig. 5:**
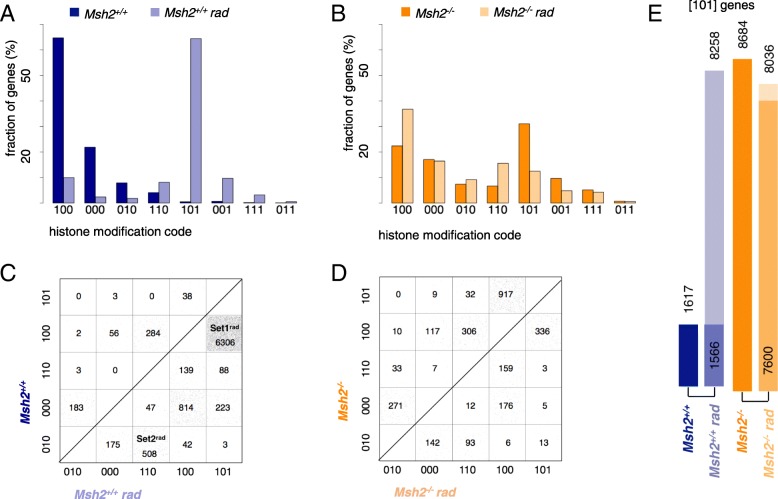
Epigenetic changes induced by a single-radiation hit. **a**, **b** The histone modification state distribution for differentially modified genes comparing non-radiated with radiated *Msh2*^*+/+*^ (**a**, 10389 genes) and *Msh2*^*−/−*^ mice (**b**, 3495 genes). **c**, **d** The transition matrices for non-radiated and radiated *Msh2*^*+/+*^ (**c**) and *Msh2*^*−/−*^ (**d**) mice. The matrix in **c** emphasizes the recruitment of H3K36me3 to histones carrying a single H3K4me3 mark (Set1^rad^) and recruitment of H3K4me3 to H3K27me3 and unmodified genes (Set2^rad^) in *Msh2*^*+/+*^ mice. The transition matrix in **d** shows no prominent enrichment comparing non-radiated and radiated *Msh2*^*−/−*^ mice. **e** Number of genes carrying an H3K4me3-H3K36me3 histone mark [101] in non-radiated mice and their radiated counterparts. The darker part of the columns of radiated mice indicates the fraction of genes that are also modified in non-radiated mice

To study the long-term epigenetic response of the intestine to radiation, we determined the histone modification profiles 4 weeks after a single-radiation hit of 0.5 Gy. The radiation hit induced phosphorylation of p53 at Ser15 (Additional file 4), a common marker of double-strand break response [33]. We found 10,389 differentially modified genes in radiated *Msh2*^*+/+*^ mice (Fig. 5a). Most of these genes either recruit H3K36me3 to H3K4me3 target genes (100 → 101, 6306 genes; Set1^rad^) or H3K4me3 to H3K27me3 target genes (010 → 110, 508 genes, Set2^rad^). In addition, unmodified genes recruit H3K4me3 (000 → 100, 814 genes) (Fig. 5b).

Surprisingly, radiation of *Msh2*^−/−^ mice induces fewer changes (3495 genes, Fig. 5c) without a prominent state transition (Fig. 5d). We confirmed the histone modification states for selected genes of untreated and radiated mice using ChIP-qPCR (Additional file 3).

The average expression of Set1^rad^ genes was not increased in radiated *Msh2*^*+/+*^ compared to untreated *Msh2*^*+/+*^ mice. Similar to Set1^−/−^ genes, a higher expression of Set1^rad^ genes was detected compared to [100] modified genes that remain [100] modified in radiated mice (Additional file 4).

### A common epigenetic response

Counting the number of genes with H3K4me3-H3K36me3 histone marks [101] in all mice (Fig. 5e), we found similar numbers of [101] modified genes in radiated *Msh2*^+/+^ (8258) and *Msh2*^−/−^ (8036) mice. Moreover, 7600 out of the 8036 [101] modified genes observed in radiated *Msh2*^−/−^ mice are already [101] modified in untreated *Msh2*^−/−^ mice. Together, these results suggest that *Msh2* loss and a single-radiation hit cause a common epigenetic response.

**Fig. 6:**
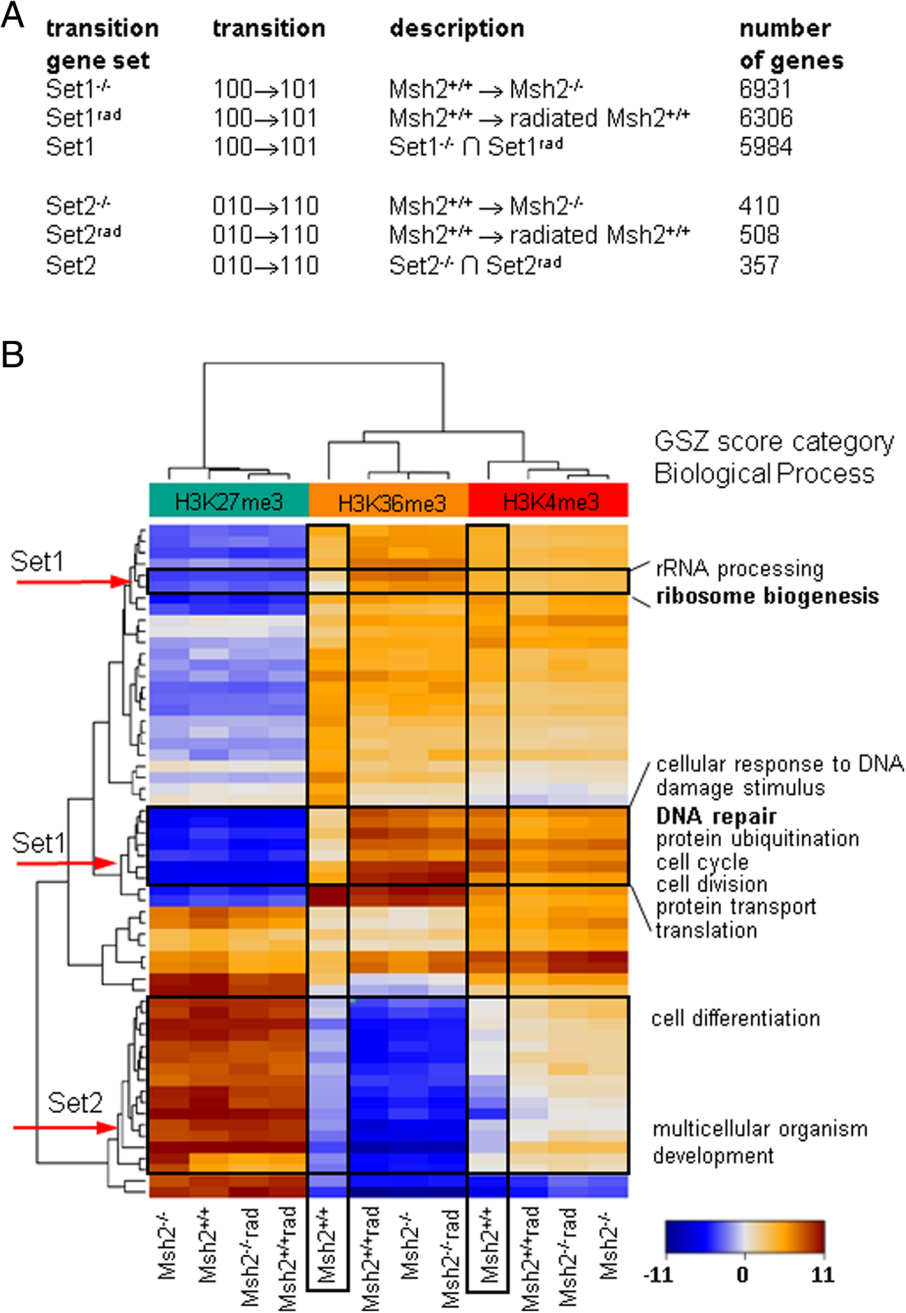
Features of the common epigenetic response. **a** Epigenetic transition gene sets. The individual sets are given together with their intersection (∩) and the numbers of genes belonging to them. **b** Heatmap of the Gene Set *Z*-scoring function (GSZ score) of modification for selected gene sets. These sets are enriched by genes contributing to the overexpression spots of the SOM calculated using the histone modification data (see Additional file 5). Entire sets are dominated by Set1 or Set2 genes (red arrows)

Focusing on genes which belong to Set1, which acquire H3K36me3 marks at promoters already modified by H3K4me3, we found that after *Msh2* loss or radiation a large gene set is affected under both conditions (Fig. 6a). In fact, 5984 genes are part of both Set1 (100 → 101) transition sets. Similarly, we found 357 genes to be part of both Set2 (010 → 110) transition sets. Notably, for a small set of genes, radiation-specific epigenetic activation is seen. Details are provided in the Additional file 5.

### Functional annotation of the common epigenetic response

To test whether Set1 and Set2 genes are associated with functional gene sets, we performed a gene ontology (GO) enrichment analysis [26, 34]. In Set1, we found enrichment in gene sets linked, among others, to ribosomal biogenesis, translation, RNA metabolic processes, mitotic cell cycle, DNA damage checkpoints, and repair mechanisms (Additional file 6). These are enrichments typically found for H3K4me3 target genes, i.e. highly expressed genes. The genes of Set2 enrich in gene sets linked to developmental processes, cell differentiation, and fate commitment, as expected for H3K27me3 target genes (Additional file 6) [35]. Among these genes are many *Wnt*, *Fox*, *Sox*, *Pax*, and *Fgf* family members and components of receptor and channel complexes. A heatmap using a Gene Set Z-scoring function (GSZ score) of modification [28] demonstrates that both Set1 and Set2 genes dominate entire functional gene sets (Fig. 6b). Details on selected gene sets are provided in the Additional file 5.

**Fig. 7:**
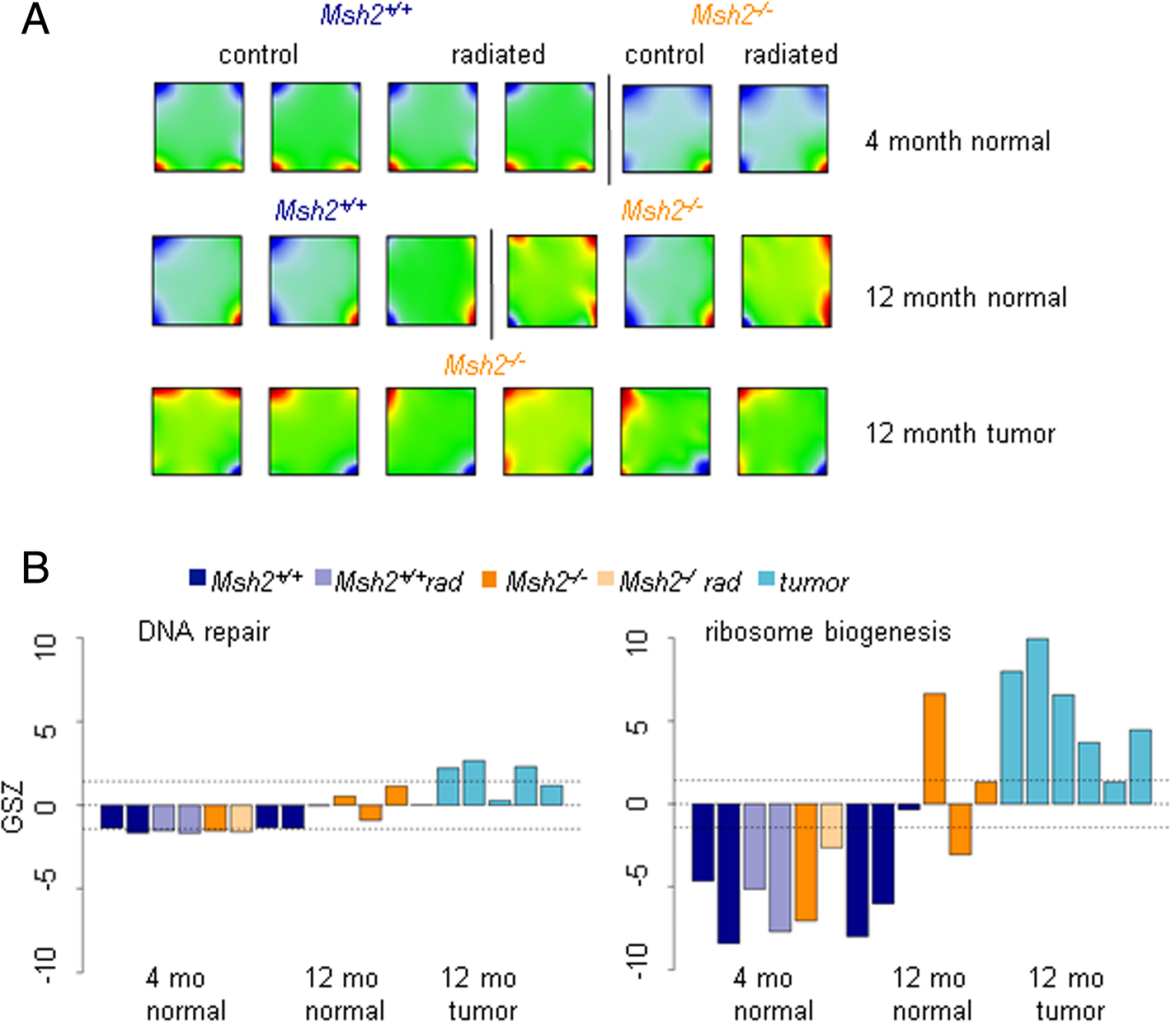
SOM-analysis of gene expression data. **a** SOM-portraits of gene expression in normal intestinal samples of untreated or radiated 4- and 12-month-old mice and tumors of 12-month-old *Msh2*^*−/−*^ mice. Color codes for gene expression which was normalized to the maximum (red) and minimum (blue) expression within the individual sample. **b** GSZ score of expression for the “DNA repair” and “ribosome” gene sets. Following the loss of *Msh2* or a single-radiation hit, the epigenetic profile of these sets is dominated by Set1 genes. (Fig. 6b, Additional file 5). An increased variability of the expression is seen in 12- compared to 4-month-old mice. Gene expression is strongly upregulated in tumors

Subsequently, we analyzed, whether the gene sets dominated by Set1 and Set2 genes are transcriptionally regulated in the macroscopic normal intestine during aging and tumorigenesis. For this purpose a SOM-analysis was performed based on gene expression data of 4- and 12-month-old mice. The data of the older mice including tumor data have been published recently [7]. Comparing the SOM portraits, a differential activation of genes in 4- and 12-month-old mice and tumors is observed (Fig. 7). Actually, several of the gene sets that are dominated by Set1 and Set2 genes are among those becoming upregulated in tumors. This is exemplified for the genes of the biological process (BP) set “DNA repair” and the cellular component (CC) set “ribosome biogenesis”.

### Set1 and Set2 genes can be predicted based on their molecular characteristics

The validity of the identified histone modification changes depends in particular on the epigenetic states determined in untreated control *Msh2*^+/+^ mice. Thus, we validated histone modifications of Set1 and Set2 genes in further controls by comparing our results with tissue data from the mouse ENCODE consortium [21]. As these experiments measured the histone modifications with different sensitivity, we compared equal numbers of best quality, gene-associated peaks. We found 4893 (81.9%) out of the Set1 genes to be [100] modified and 267 (74.8%) out of the Set2 genes to be [010] modified, supporting our results. In addition, we compared our data with H3K4me3 and H3K27me3 data on isolated mouse intestinal epithelial cells provided by Kazakevych et al. [22] including data on cells of embryonic days E12.5 and E14.5, ISC and adult enterocytes (AE). Modification profiles seen in ISC and AE are very similar to our tissue profiles, while those from embryonic cells deviate (Additional file 7). We conclude that the profiles identified for Set1 and Set2 genes are characteristic for absorptive cells of the adult mouse intestine.

Subsequently, we assigned Set1 and Set2 genes further molecular characteristics, reanalyzing methyl-CpG-binding domain (MBD) and RNA-seq data provided by Kazakevych et al. [22]. We found that, in isolated ISC and AE, Set1 (Set2) genes show relatively low promoter DNA methylation compared to genes [100] ([010]) modified in both untreated *Msh2*^+/+^ and *Msh2*^−/−^ mice (Additional file 8). Moreover, the genes of both sets are characterized by a specific gene body DNA methylation. As in our tissue samples, Set1 genes are higher expressed compared to genes [100] modified in both untreated *Msh2*^+/+^ and *Msh2*^−/−^ mice (Additional file 8). Among all [100] genes, the 1000 genes with the highest (lowest) expression in ISC cover 693 (119) Set1 genes which is a 1.23 (0.21)-fold enrichment (depletion). Together, these results demonstrate that Set1 and Set2 genes carry specific properties that allow their prediction based on data from control intestinal tissue.

This led us to analyze the conservation of these properties in human. We measured histone modifications in normal human colon and combined these data with published matched RNA-seq data [3]. From the 5984 Set1 mouse genes, we found 5065 (85%) to be conserved in human. Among them, 49.4% show a [100] profile as in mice but 32.4% a [101] profile (Additional file 9). Assuming a comparable sensitivity of our measurements in mouse and human, this suggests an already present epigenetic activation in human. From the 357 Set2 mouse genes, we found 253 (71%) to be conserved in human. Among them, only 28.3% show a [010] profile as in mice and 34.6% a [110] profile. Thus, an epigenetic activation of these genes might be present as well.

Similar to our observations in *Msh2*^++^ mice, expression of Set1 genes in the human colon is above the expression of those [100] genes not contributing to Set1.

## Discussion

Here, we show that macroscopic normal intestinal tissue of 4-month-old *Msh2*^*−/−*^ mice exhibit genome-wide epigenetic changes compared to age-matched control mice although intestinal tumors do not develop in *Msh2*^*−/−*^ mice until the age of 12 months [7]. Thus, tumorigenesis under MMR deficiency occurs on a disturbed epigenetic background. Comparing non-radiated with radiated *Msh2*^*+/+*^ mice, differences in the histone H3 methylation profiles are very similar to those induced by *Msh2* loss, suggesting a common epigenetic response which is most likely part of a response to genomic stress at the cell level. It is unlikely that the same large gene sets are damaged in both experimental settings, MMR deficiency, and a single-radiation hit.

Questions arise on the mechanisms controlling this common epigenetic response. Spontaneous mutation rates in MMR-deficient tissue are much higher than in control tissue. In the human colon, mismatch mutations increase immediately after the loss of *MLH1* and *MSH2* [4]. In non-conditional *Msh2*-knockout mice mutations rates at endogenous expanded simple tandem repeat (ESTR) DNA loci rise in the germline [36]. Comparable to our study, a single-radiation hit of these mice did not further increase the ESTR mutation rate, whereas a significant induction of mutations was observed in radiated controls. Burr et al. hypothesized that high cell killing in radiated MMR-deficient tissue explains the phenomenon [36]. A similar explanation might also hold true for our observed epigenetic changes. We suggest that the common epigenetic response results from a selection of ISC tolerant to genomic stress. Less tolerant ISC either undergo apoptosis or are, in agreement with experimental findings on radio-resistance of ISC, outcompeted in each individual crypt [37, 38]. This assumption consistently explains the epigenetic response in *Msh2*^*−/−*^ mice on radiation, where the selection of ISC might have already taken place. In agreement with a response to genomic stress at the cell level, a short-term epigenetic response following double-strand repair does not comprise changes in H3K36me3 [39, 40], which are obviously present 4 weeks after radiation or after *Msh2* loss.

A competitive advantage of ISC subsequent to the epigenetic activation in Set1 and Set2 genes may be explained as follows:

(1) H3K36me3 accumulates most frequently at [100] genes, i.e., genes that are H3K4me3 modified in control mice. These genes are important for ribosomal biogenesis, translation, and RNA processing. An impairment of any of these cellular processes can severely retard cell growth and perturb mammalian development [41]. H3K36me3 is required for proper recruitment of the MMR machinery [42] and homologous recombination (HR) [43], and it regulates the mode of double-strand break repair in favor of non-homologous end joining (NHEJ) [44], the preferred repair mode in the intestine [45]. There is a strong inverse correlation between normalized H3K36me3 level and mutational frequency [46]. Thus, higher H3K36me3 at Set1 genes might ensure higher fidelity of ISC. In the absence of Setd2 and thus of H3K36me3, the capacity for clonogenic survival following DNA damage is significantly reduced [39].

(2) Accumulation of H3K4me3 was predominantly observed for [010] genes, i.e., genes that are repressed by H3K27me3 in control mice. Among these genes are many that control developmental processes and differentiation [25]. Upregulation of such genes might be important for responding to regenerative demands [47]. Here, the recruitment of H3K4me3 in Set2 genes might represent a mechanism to protect their promoters from DNA methylation and thus to ensure potential gene activation according to the following mechanism: DNA damage triggers promoter DNA methylation of H3K27me3 target genes [48]. Recruitment of H3K4me3 to H3K27me3-labeled gene loci can prevent DNA methylation in *Msh2*^*−/−*^ mice [49]. Consistently, loss of *Msh2* prevented promotor CpG island hypermethylation observed in inflammation-induced tumors [50].

Based on our data, we cannot exclude that the epigenetic response is an intrinsic regulation within all cells. Moreover, it remains unclear, how the enrichment of activating histone methylation marks is achieved and stabilized. We did not find an increased gene expression paralleling the broad H3K36me3 enrichment. Thus, changes in the activity of histone methyltransferases and/or demethylases of H3K36 are likely.

Until now, the epigenetic response following DNA damage has been predominantly studied within a short-term window (reviewed in [51, 52]). Here, we provide evidence that loss of *Msh2* and a single-radiation hit induce common epigenetic changes that persist for long time scales. We expect that the advantages of an epigenetic activation of the affected gene sets are accessed also by tumor cells. *Msh2*^*−/−*^ tumors show a transcriptional activation of many of the affected genes [7]. This suggests that the observed epigenetic changes protect DNA loci encoding essential cellular functions also in tumor cells. Indeed, tumorigenesis is often controlled by regulating ribosome biogenesis and global protein synthesis [41]. For about half of the Set1 genes, histone profiles and relative transcription levels seen in control mice are conserved in human. Thus, we expect similar response scenarios also in the human intestine. In fact, mismatch repair-deficient Lynch colorectal carcinoma with high mutational load show overexpression of gene sets associated with the GO gene sets “translation,” “ribosome,” and “cell cycle” [3] which we found to be associated with Set1 genes. It would be interesting to see whether targeting H3K36me3 by SetD2 inhibitors selectively affects tumor cell survival in Lynch colorectal carcinoma.

In summary, MMR deficiency and a single radiation hit result in common genome-wide changes of histone H3 methylation profiles.

## Additional files

Additional file 1: Primer sequences. List of all primers used in the study. (XLSX 10 kb)

Additional file 2: Peak characteristics of histone methylations of mouse intestinal samples [54]. (DOCX 543 kb)

Additional file 3: ChIP-qPCR for selected genes. (DOCX 114 kb)

Additional file 4: Details on the radiation response [33]. (DOCX 196 kb)

Additional file 5: Results of the SOM analysis of the epigenetic profiles. SOM portraits and additional information about affected gene sets. (DOCX 389 kb)

Additional file 6: Results of the GO-analysis for Set1 and Set2 genes. (XLSX 15 kb)

Additional file 7: Comparison of epigenetic states in intestinal tissue and isolated intestinal cells [22]. (DOCX 515 kb)

Additional file 8: Molecular characterization of Set1 and Set2 genes in isolated intestinal cells. Published MBD- and RNA-seq data on Set1 and Set2 genes [22]. (DOCX 73 kb)

Additional file 9: Set1 and Set2 genes in mouse and human intestinal tissue. Comparison of Set1 and Set2 genes in mouse and human tissue [3]. (DOCX 888 kb)

Additional file 10: Modification profiles of human genes. The list includes those genes that show a modification in at least one of the lysines K4, K27 or K36 of histone 3 only. (XLSX 3040 kb)

## Abbreviations

AE: Adult enterocytes
ChIP: Chromatin immunoprecipitation
ISC: Intestinal stem cell
MMR: Mismatch repair
MSI: Microsatellite instability
SOM: Self-organizing map
