# Supplementary material for: Loss of Msh2 and a single-radiation hit induce common, genome-wide, and persistent epigenetic changes in the intestine
Source: Clin Epigenetics. 2019 Apr 27;11:65. doi: 10.1186/s13148-019-0639-8 (PMC6486978; doi:10.1186/s13148-019-0639-8)
Supplement: Supplementary file 2 — Peak characteristics of histone methylations of mouse intestinal samples [54]. (DOCX 543 kb) [file 13148_2019_639_MOESM2_ESM.docx]

**Additional file 2**

**Peak characteristics of histone methylations of mouse intestinal samples**


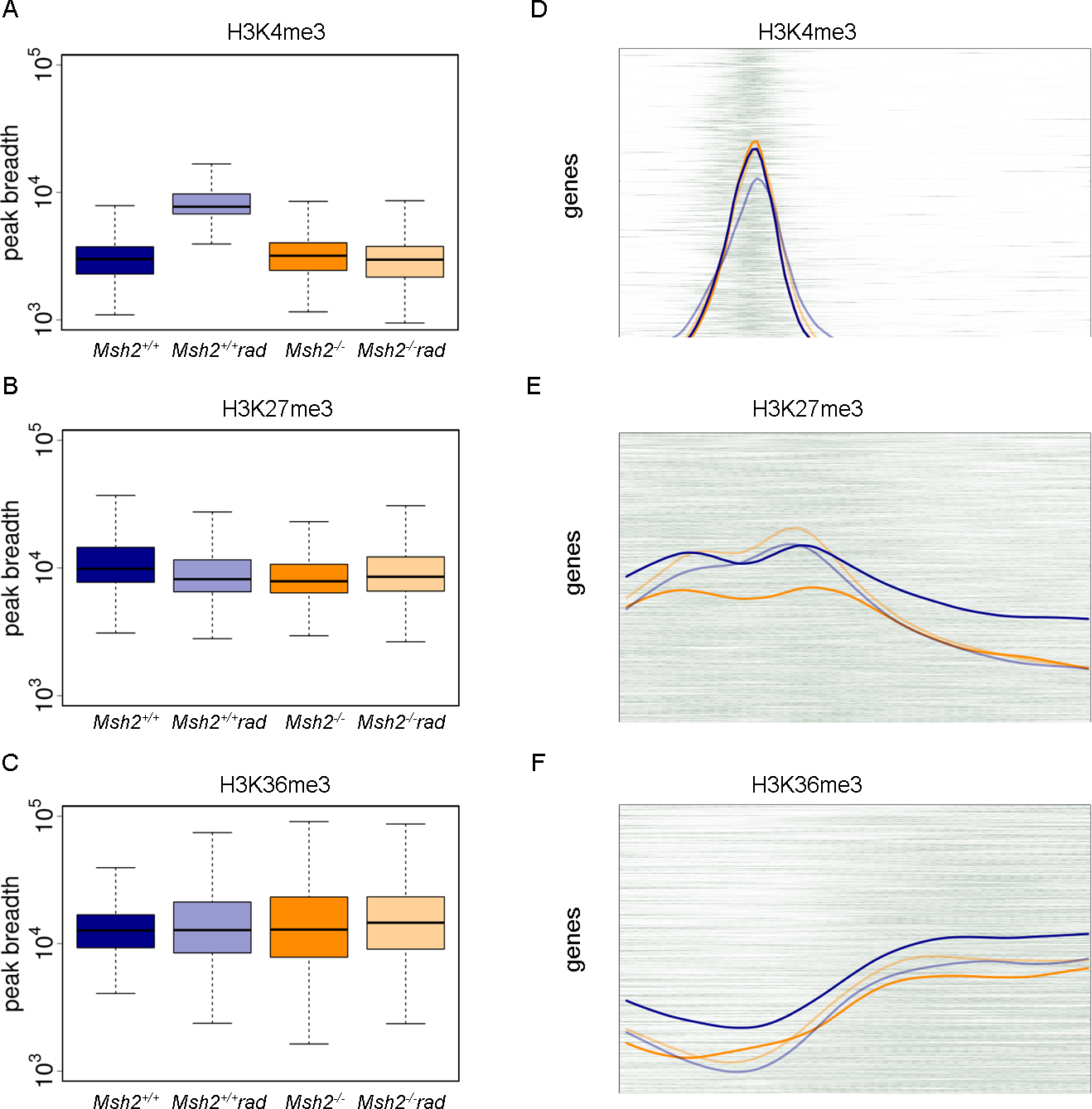


**Figure AF2: Peak characteristics of histone methylations of mouse intestinal samples**

A-C) Boxplots of peak breadths for all intestinal samples (n=2/genotype/treatment). The breadth distributions of the modification peaks are rather broad ranging from a few hundred to several thousand base pairs, except for radiated *Msh2*^+/+^ mice, which show very broad peaks (A). In *Msh2^+/+^* mice H3K27me3 regions are slightly broader compared to all other mice mice (B). Notably, the variability of H3K36me3 peak breadths is lower in *Msh2^+/+^* compared to all other mice (C).

D-F) Heatmaps showing the normalized read density for the histone modifications. Herein, each row corresponds to one gene and each column to a 100 bp window with a position relative to the transcription start site (TSS). The background color indicates the normalized read density at this position for *Msh2^+/+^* mice. Lines illustrate the mean read densities. H3K4me3 read densities are sharp and peak next to the TSS (D). In contrast, the H3K27me3 densities show a bimodal distribution (E). In agreement with ChIP-seq measurements in other stem cell systems [54], H3K36me3 read densities are low near the TSS, but establish broad peaks in the gene body (F).
