# Supplementary material for: Loss of Msh2 and a single-radiation hit induce common, genome-wide, and persistent epigenetic changes in the intestine
Source: Clin Epigenetics. 2019 Apr 27;11:65. doi: 10.1186/s13148-019-0639-8 (PMC6486978; doi:10.1186/s13148-019-0639-8)
Supplement: Supplementary file 3 — ChIP-qPCR for selected genes. (DOCX 114 kb) [file 13148_2019_639_MOESM3_ESM.docx]

**Additional file 3**

**ChIP-qPCR for selected genes**

**
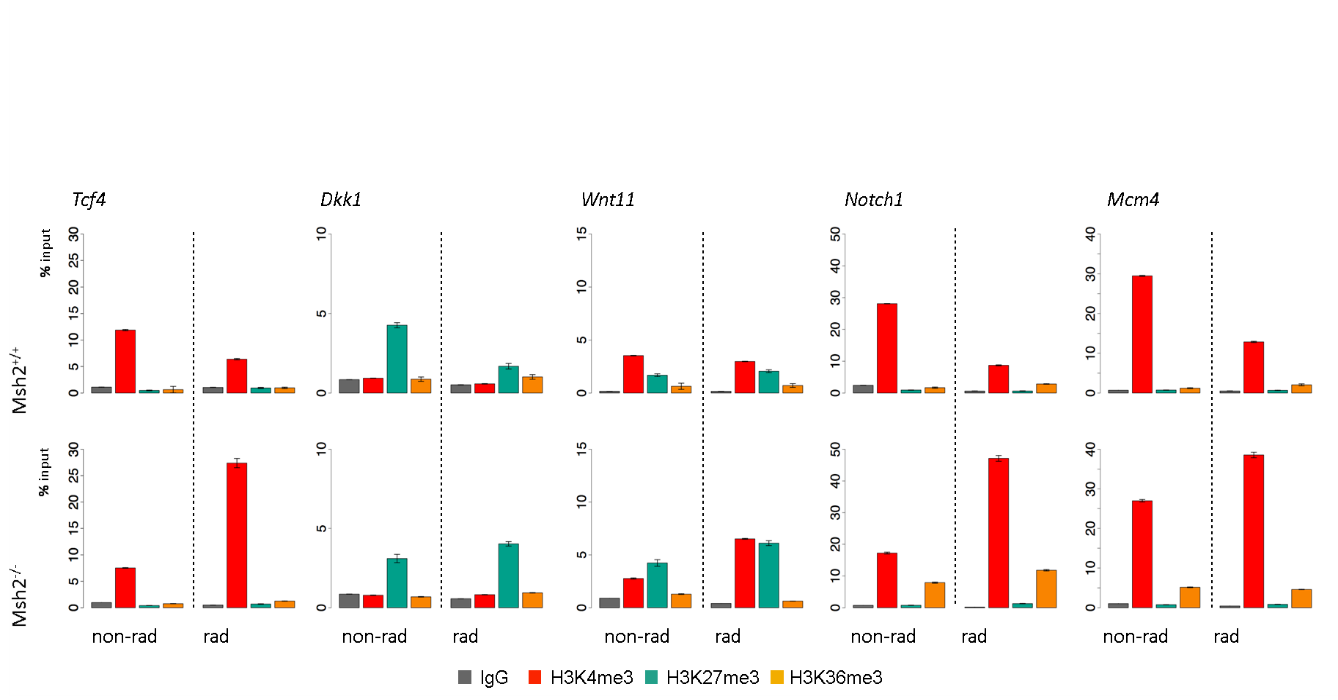
**

**Figure AF3: ChIP-qPCR for selected genes**. The histone modification peaks obtained by ChIP-seq have been validated for selected genes by ChIP-qPCR. Analysis was performed on replicates of non-radiated and radiated *Msh2^+/+^* *and Msh2^-/-^* mice. All PCR results were normalized to input controls and to an unmodified gene region (mean ± SD, n=4).
