# Supplementary material for: Loss of Msh2 and a single-radiation hit induce common, genome-wide, and persistent epigenetic changes in the intestine
Source: Clin Epigenetics. 2019 Apr 27;11:65. doi: 10.1186/s13148-019-0639-8 (PMC6486978; doi:10.1186/s13148-019-0639-8)
Supplement: Supplementary file 4 — Details on the radiation response [33]. (DOCX 196 kb) [file 13148_2019_639_MOESM4_ESM.docx]

**Additional file 4**

**Details on the radiation response**

**Figure AF4. Details on the radiation response**

A) To demonstrate DNA damage following radiation, we determined phospho-p53 [33]. Proteins were prepared from the jejunum of *Msh2*^+/+^ mice 1h after radiation with the indicated doses. 200 µg of protein were loaded per lane and analyzed with phospho-p53 (Cell Signalling, Leiden, The Netherlands) and β-actin (Abcam, Cambridge, UK) antibodies by Western Blotting. Phosphorylation of p53 at Ser15 indicates radiation-induced DNA damage**.** Mr, molecular weight marker (kDa); ctr, untreated control mouse.

B) Boxplot of the average expression of Set1^rad^ genes. Similar to Set1^-/-^ genes, the genes show an intermediate expression between stable [100] and stable [101] modified genes. While radiation induces persistent changes in histone methylation profiles, a long-term transcriptional response was not seen, in particular not in Set1^rad^ genes.
