# Supplementary material for: Loss of Msh2 and a single-radiation hit induce common, genome-wide, and persistent epigenetic changes in the intestine
Source: Clin Epigenetics. 2019 Apr 27;11:65. doi: 10.1186/s13148-019-0639-8 (PMC6486978; doi:10.1186/s13148-019-0639-8)
Supplement: Supplementary file 5 — Results of the SOM analysis of the epigenetic profiles. SOM portraits and additional information about affected gene sets. (DOCX 389 kb) [file 13148_2019_639_MOESM5_ESM.docx]

**Additional file 5**

**
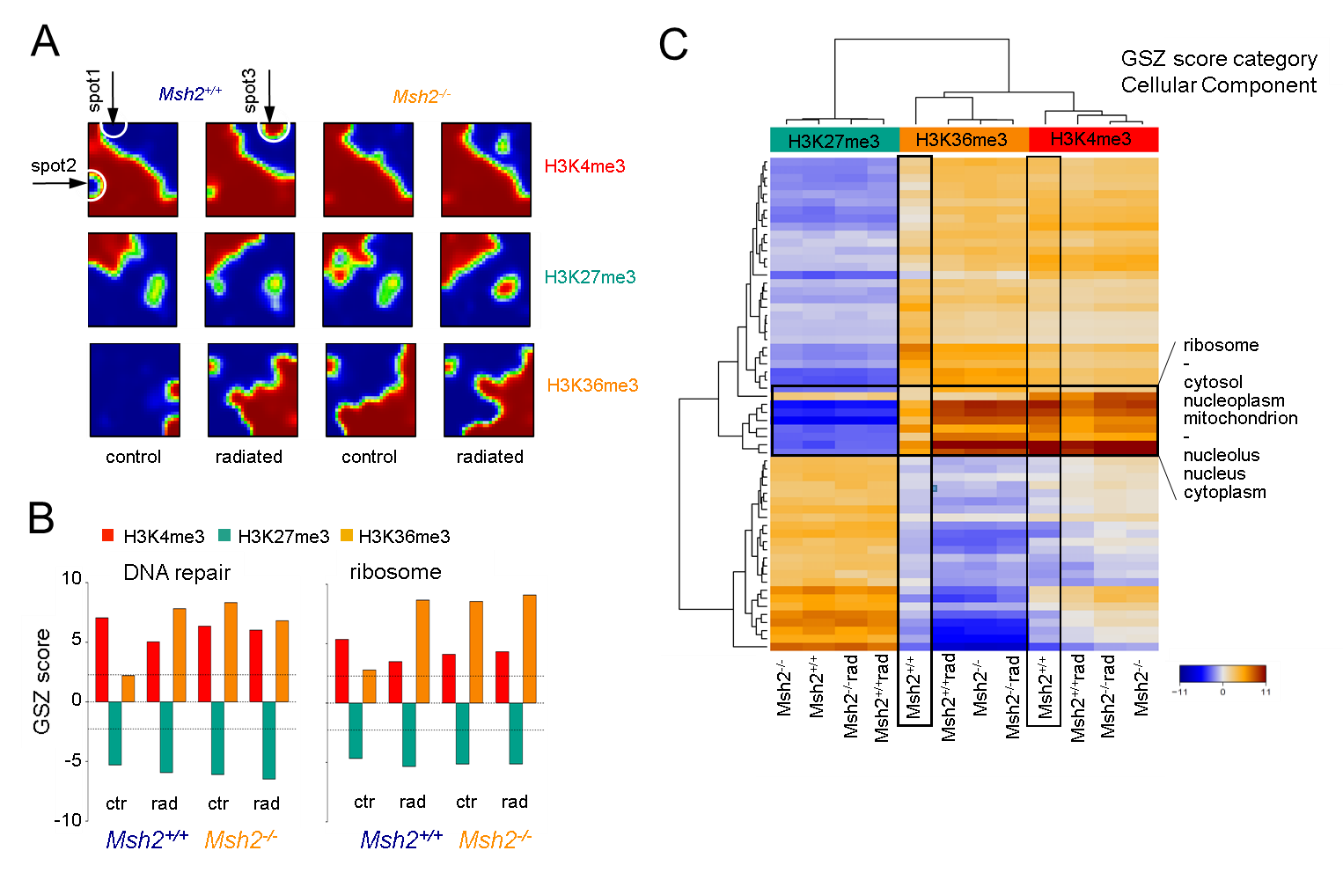
Results of the SOM analysis of the epigenetic profiles**

**Figure AF5. SOM-analysis for histone methylation profiles.**

A) SOM-portraits of all histone modification samples. They represent a fingerprint of their epigenetic regulation and thus enable a straightforward overview of the differences between the modification profiles. The common epigenetic response is documented by the extension of H3K4me3 (e.g. spot 1 and spot 2) and in particular of H3K36me3 modification spots. A radiation-specific H3K4me3 spot (spot 3) is observed comprising unmodified genes that recruit H3K4me3 after radiation in *Msh2^+/+^* mice only.

B) GSZ score of modification for all genes of the GO set ‘Biological Process (BP): DNA repair’ and those of GO set ‘Cellular Component: ribosome’. The common epigenetic response dominates the modification profiles of both gene sets.

C) Heatmap of the GSZ score of modification for ‘Cellular Component’ gene sets. As for the heatmap shown in Fig. 6, gene sets have been selected that are enriched by genes contributing to the overexpression spots of the SOM portraits. Entire sets (indicated) increase H3K36me3 following *Msh2* loss or a single radiation hit. Thereby, weak H3K36me3 modification in *Msh2^+/+^* mice, as characteristic for Set1 genes, is seen for genes of the ‘ribosome’ and ‘nucleolus’ gene set, only.
