# Supplementary material for: Loss of Msh2 and a single-radiation hit induce common, genome-wide, and persistent epigenetic changes in the intestine
Source: Clin Epigenetics. 2019 Apr 27;11:65. doi: 10.1186/s13148-019-0639-8 (PMC6486978; doi:10.1186/s13148-019-0639-8)
Supplement: Supplementary file 7 — Comparison of epigenetic states in intestinal tissue and isolated intestinal cells [22]. (DOCX 515 kb) [file 13148_2019_639_MOESM7_ESM.docx]

**Additional file 7**

**Comparison of epigenetic states in intestinal tissue and isolated intestinal cells.**

**
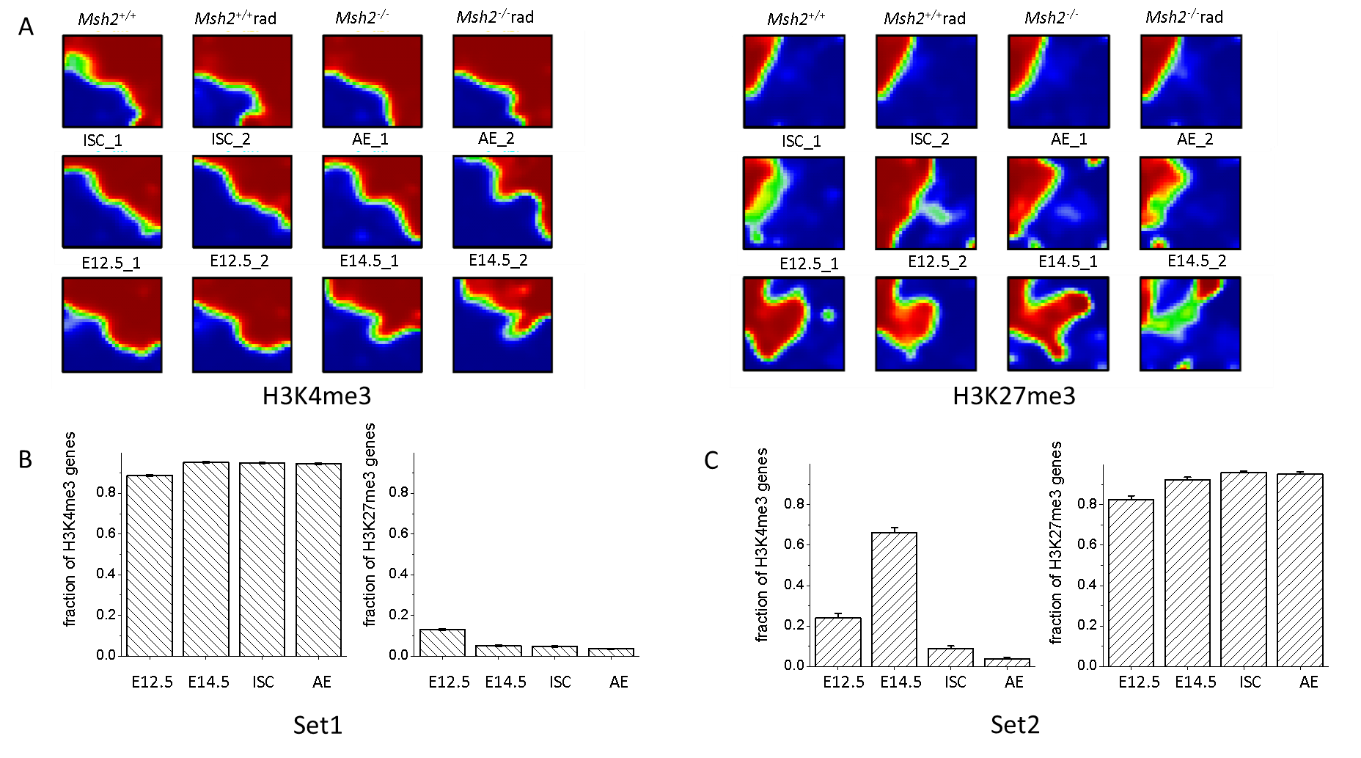
**

**Figure AF7. Comparison of epigenetic states in intestinal tissue and isolated intestinal cells.**

A) SOM-portraits of H3K4me3 and H3K27me3 in intestinal tissue of the various radiated and untreated *Msh2* mice and isolated mouse embryonic intestinal cells at days E12.5 and E14.5, intestinal stem cells (ISC) as well as adult enterocytes (AE); provided by [22]. Portraits of ISC and AE are similar to the tissue, while those of embryonic cells deviate.

B/C) Histone modifications of Set1 (B) and Set2 (C) genes as seen in the data provided by [22]. Shown are the fractions of genes carrying the respective modification. Averages are taken over samples of the same origin.

B) More than 94% of the Set1 genes are H3K4me3 modified and less than 5% are H3K27me3 modified in ISC and AE in agreement with our tissue data. Errors: SE, n= 5411 genes (90.4% of Set1)

C) More than 95% of the Set2 genes are H3K27me3 modified and less than 9% are H3K4me3 modified in ISC and AE in agreement with our tissue data. Errors: SE, n=290 genes (81.2% of Set2).
