# Supplementary material for: Loss of Msh2 and a single-radiation hit induce common, genome-wide, and persistent epigenetic changes in the intestine
Source: Clin Epigenetics. 2019 Apr 27;11:65. doi: 10.1186/s13148-019-0639-8 (PMC6486978; doi:10.1186/s13148-019-0639-8)
Supplement: Supplementary file 8 — Molecular characterization of Set1 and Set2 genes in isolated intestinal cells. Published MBD- and RNA-seq data on Set1 and Set2 genes [22]. (DOCX 73 kb) [file 13148_2019_639_MOESM8_ESM.docx]

**Additional file 8**

**Molecular characterization of Set1 and Set2 genes in isolated intestinal cells.**

**
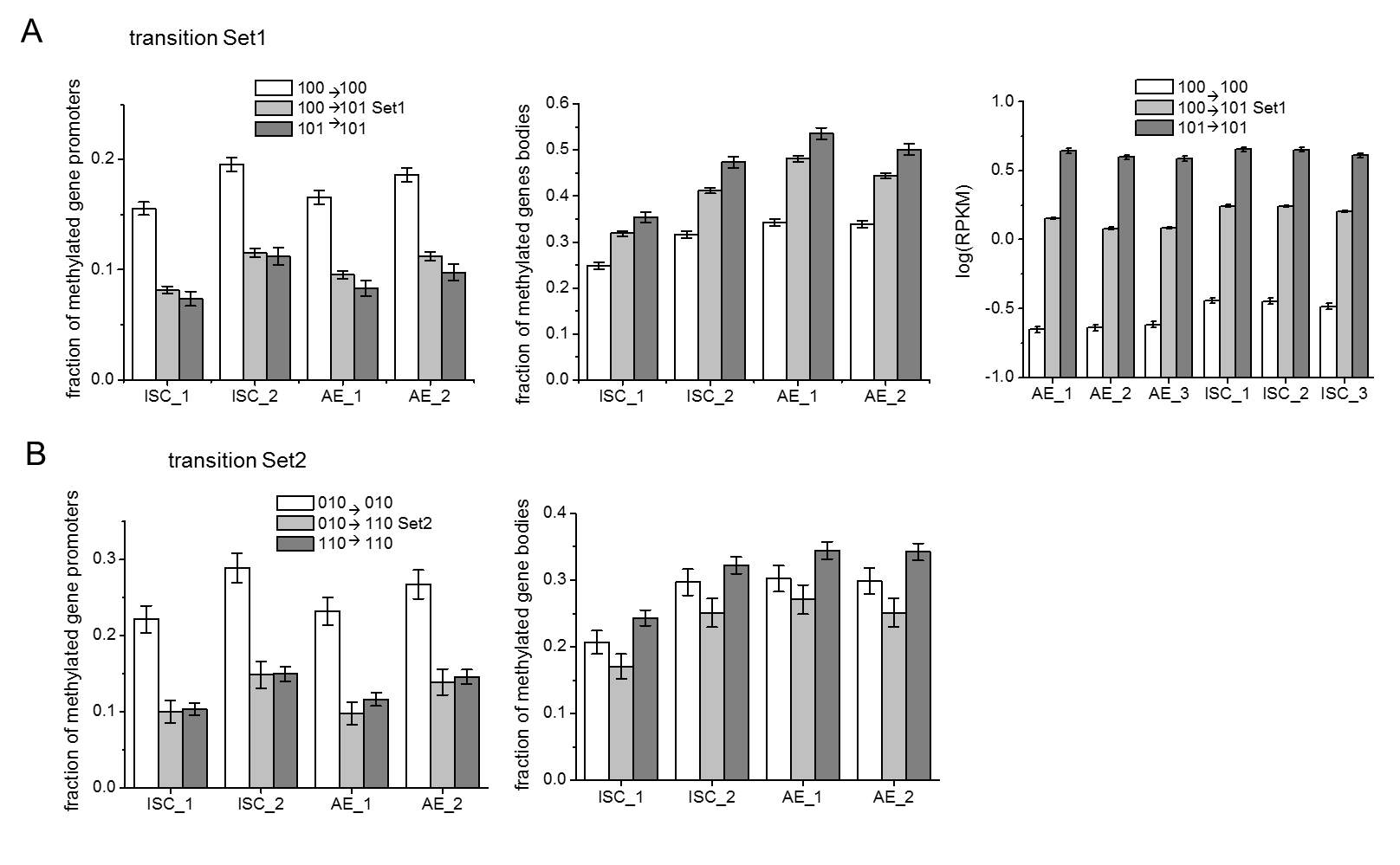
**

**Figure AF8.** **Molecular characterization of Set1 and Set2 genes**

RNA-seq and Methyl-seq data of isolated ISCs and adult enterocytes (AE), provided by [22], were reanalyzed to extend the molecular characteristics of transition Set1^-/-^ and Set2^-/-^ genes. Errors: SE

A) Properties of H3K4me3 modified genes that recruit H3K36me3 in *Msh2^-/-^* mice (i.e. Set1^-/-^ genes). Set1^-/-^ and stable [101] genes show lower promoter and higher gene body methylation compared to stable [100] genes. Their expression, given in reads per kilobase million (RPKM), is higher than that of stable [100] genes.

B) DNA methylation of H3K27me3 modified genes that recruit H3K4me3 in *Msh2^-/-^* mice (i.e. Set2^-/-^ genes). Set2^-/-^ and stable [110] genes show much lower promoter DNA methylation compared to stable [010] genes, while the gene body DNA methylation of all these genes is comparable.
