# Supplementary material for: Loss of Msh2 and a single-radiation hit induce common, genome-wide, and persistent epigenetic changes in the intestine
Source: Clin Epigenetics. 2019 Apr 27;11:65. doi: 10.1186/s13148-019-0639-8 (PMC6486978; doi:10.1186/s13148-019-0639-8)
Supplement: Supplementary file 9 — Set1 and Set2 genes in mouse and human intestinal tissue. Comparison of Set1 and Set2 genes in mouse and human tissue [3]. (DOCX 888 kb) [file 13148_2019_639_MOESM9_ESM.docx]

**Additional file 9**

**Set1 and Set2 genes in mouse and human intestinal tissue.**

**Figure AF9. Set1 and Set2 genes in mouse and human intestinal tissue.**

A) SOM-portraits of mouse intestinal tissue and data obtained from a normal human colon [3]. The portraits of human and mouse intestinal tissue are similar. The SOM portraits comprise modification states of 13,576 genes conserved between human and mice.

B) Distribution of histone modification profiles throughout Set1 and Set2 genes conserved in human. About half of these Set1 genes but less than one third of these Set2 genes conserve their profile.

C) RPKM values obtained for Set1 genes being conserved and [100] or [101] modified in human. They are compared with RPKM values of genes being [100] modified in human but do not contribute to the Set1 gene set. Genes of both Set1 subsets are significantly higher expressed (p-value<0.001).
